# *TomoPyUI*: a user-friendly tool for rapid tomography alignment and reconstruction

**DOI:** 10.1107/S1600577524003989

**Published:** 2024-06-26

**Authors:** Samuel S. Welborn, Molleigh B. Preefer, Johanna Nelson Weker

**Affiliations:** a Department of Materials Science and Engineering University of Pennsylvania Philadelphia PA19104 USA; b Stanford Synchrotron Radiation Lightsource SLAC National Accelerator Laboratory Menlo Park CA94025 USA; University of Malaga, Spain

**Keywords:** *TomoPyUI*, computed tomography, alignment, reconstruction, *TomoPy*, *Jupyter*

## Abstract

The management and processing of synchrotron and neutron computed tomography data can be a complex, labor-intensive and unstructured process. Users devote substantial time to both manually processing their data (*i.e.* organizing data/metadata, applying image filters *etc.*) and waiting for the computation of iterative alignment and reconstruction algorithms to finish. In this work, we present a solution to these problems: *TomoPyUI*, a user interface for the well known tomography data processing package *TomoPy*. This highly visual Python software package guides the user through the tomography processing pipeline from data import, preprocessing, alignment and finally to 3D volume reconstruction. The *TomoPyUI* systematic intermediate data and metadata storage system improves organization, and the inspection and manipulation tools (built within the application) help to avoid interrupted workflows. Notably, *TomoPyUI* operates entirely within a *Jupyter* environment. Herein, we provide a summary of these key features of *TomoPyUI*, along with an overview of the tomography processing pipeline, a discussion of the landscape of existing tomography processing software and the purpose of *TomoPyUI*, and a demonstration of its capabilities for real tomography data collected at SSRL beamline 6-2c.

## Abstract 2

In this study, we introduce *TomoPyUI*, a user-friendly interface for the *TomoPy* tomography data processing package, designed to streamline and enhance the management of synchrotron and neutron computed tomography data. *TomoPyUI*, operating within a *Jupyter* environment, incorporates GPU-accelerated algorithms and a structured data management system, significantly reducing computation times and improving workflow efficiency in the tomography processing pipeline.

## 1. Introduction

During time-sensitive experiments, users would like to rapidly process and assess their data directly after acquisition to draw immediate conclusions from the data, which can dictate their next experimental step and save limited beam time. Further, it allows users to finish their beam time with processed data. Unfortunately, data processing is frequently held up by a complicated, time-consuming data processing pipeline that may be unfamiliar to the user, especially an inexperienced one. Navigating this complexity frequently necessitates guidance from experienced personnel, imposing additional demands on beamline staff. Further, both the experimental settings and the sample type impact ease of processing. User-friendly tools that provide an intuitive and interactive workspace for data visualization and processing should therefore be highly valued. By reducing time spent teaching and learning the assemblage of software, we can reduce the time between data acquisition and scientific understanding.

In this work, we aim to improve the experience of synchrotron and neutron computed tomography (CT) users by enhancing *TomoPy* (Gürsoy *et al.*, 2014 ▸; Pelt *et al.*, 2016 ▸), a popular text-based software package for tomography data processing, to *TomoPyUI* (https://github.com/swelborn/tomopyui), a graphical user interface (GUI) that prioritizes visualization, interactivity, and standardized data and metadata storage. Here we provide background on tomography data processing and currently available software, introduce the purpose of *TomoPyUI*, discuss its key features, present the results obtained from using it to align and reconstruct a real dataset, and provide details on its underlying code structure to facilitate its uptake at other user facilities. Although the modules in *TomoPyUI* are focused on processing and visualizing tomography datasets, we believe the logic behind its primary features are generalizable to other types of image series data.

## 2. Background

### 2.1. Computed tomography processing

In the age of *in situ* and *operando* tomography and high-brightness synchrotrons, hundreds of tomography datasets can be collected in a given beam time. This leaves the user with potentially thousands of projection images of the sample to preprocess, align and finally reconstruct into a 3D volume representation using a selection of available reconstruction algorithms. The data processing pipeline requires several steps: preprocessing data, searching for the center of rotation, projection image alignment and reconstruction.

(1) *Preprocessing data.* Reference and dark images before, during and/or after an acquisition are used to correct for background signal present in the projection images (normalization). Multiple exposures of reference/dark images and projection images may be collected to avoid detector saturation and reduce the signal-to-noise ratio, in which case they need to be averaged together. After normalization and averaging, it may be necessary to apply preprocessing such as image filtering and destriping to minimize artifacts in the reconstructed image (Vo *et al.*, 2018 ▸).

(2) *Searching for the center of rotation*. The center of rotation (the axis around which the sample rotates) typically lies at some offset from the center pixel of the projection images due to the inherent challenge of aligning the sample perfectly at the time of data acquisition. Finding the correct center of rotation is critical, as severe artifacts will appear in the reconstruction if it is set incorrectly (Gürsoy *et al.*, 2014 ▸; Yang *et al.*, 2015 ▸). Further, if the rotation axis is not exactly perpendicular with respect to the bottom of the projection image, it will vary along the height of the images.

(3) *Projection image alignment.* High-resolution tomography data can suffer from jitter – imprecision of the rotation motor sample stage (runout) causes the sample to jump around in the field-of-view during data collection (Gürsoy *et al.*, 2017 ▸; Wang, 2020 ▸). This motion is especially severe during the collection of X-ray nanotomography data (Gürsoy *et al.*, 2017 ▸; Wang, 2020 ▸). Solutions to this problem vary and include the use of external sensors to correct for misalignments or automatic alignment algorithms to adjust the raw data (Gürsoy *et al.*, 2017 ▸; Wang, 2020 ▸; Stankevič *et al.*, 2017 ▸; Wang *et al.*, 2012 ▸; Liu *et al.*, 2012 ▸; Larsson *et al.*, 2019 ▸; Pande *et al.*, 2022 ▸; Nikitin *et al.*, 2021 ▸). Often, automatic alignments do not work as expected, and users resort to manually aligning the raw data in a user interface.

(4) *Reconstruction*. A number of reconstruction algorithms, which fall into the category of either analytic or iterative algorithms, can be used to perform a transformation from the projection image domain to the reconstructed image domain (Withers *et al.*, 2021 ▸). The details of these algorithms are beyond the scope of this work, but can be found in review articles such as Withers *et al.* (2021 ▸).

### 2.2. Tomography processing packages

Many software packages exist to aid in the reconstruction of tomography data, including GUIs (Liu *et al.*, 2012 ▸; Xiao *et al.*, 2022 ▸; Pandolfi *et al.*, 2018 ▸) and text-based applications (Gürsoy *et al.*, 2014 ▸; Pelt *et al.*, 2016 ▸; Wadeson & Basham, 2016 ▸; Vo *et al.*, 2019 ▸, 2021 ▸; van Aarle *et al.*, 2016 ▸) which implement the full processing pipeline. While we discuss some of these tools in this work, a more exhaustive list can be found on the repository at https://tomopedia.github.io/software/. TXM *Wizard* (Liu *et al.*, 2012 ▸) is a GUI based on *Matlab* capable of each of the main tomography processing steps (and more), but is not open-source. This makes the addition of any novel and more robust alignment or reconstruction routines difficult; it would require source access and a *Matlab* license. *TXM-Sandbox* is a recently developed GUI that uses both *TomoPy* and *ImageJ* for external image processing and analysis (Xiao *et al.*, 2022 ▸). While *ImageJ* offers a wide variety of image processing tools, moving data into another application can decentralize the workflow unless it is integrated directly into the application like in *AreaDetector* (Rivers *et al.*, 2010 ▸) or *TomoStream* (Nikitin *et al.*, 2022 ▸). *Tomviz* (Schwartz *et al.*, 2022 ▸), a GUI developed by *Kitware*, provides a robust graphical interface for rendering, manipulating and analyzing 3D tomograms. It is capable of handling the full pipeline of data processing steps from reconstruction to visualization to analysis of 3D data, with Python tools for 3D analysis.

Workflow management tools enable users to create a series of steps that can be represented in a directed acyclic graph (DAG) and then execute this graph. *Tomwer* enables workflow automation by tailoring the *ESRF Workflow System* (*ewoks*) to NXTomo datasets (Payno *et al.*, 2022 ▸). Tasks can be drawn on a canvas and set up to automate data processing as scans are acquired. Further, it integrates with the *slurm* scheduler, making it possible to run workflows on remote resources. *Tomwer* can be used alongside *nabu*, the ESRF command line interface workflow tool (Paleo *et al.*, 2019 ▸). *Tofu* (Faragó *et al.*, 2022 ▸) is a workflow toolkit that utilizes the *ufo framework* (Albarghouthi *et al.*, 2012 ▸) for rapid GPU processing. Similar to *tomwer*, it enables users to create a workflow graph in a user interface, and is optimized for use on a single workstation. On the other hand, the workflow tool *Savu*, in operation at Diamond Light Source, has the ability to ingest a list of commands and run them on a compute cluster, making use of the *Message Passing Interface* (*MPI*) to run workflows in parallel (Wadeson & Basham, 2016 ▸).

In the domain of text-based tools, command line interfaces (CLI) such as *tomopy-cli* (Gürsoy *et al.*, 2014 ▸) offer a streamlined solution for users seeking to quickly and efficiently reconstruct tomography data directly within a terminal environment. This approach is advantageous for batch operations where processing parameters remain constant across datasets, and eliminates the need for script editing. Users can execute reconstruction commands by specifying parameters at runtime, or by using configuration files.

The majority of the solutions discussed above adhere to a post-acquisition model, where data are initially stored on disk before being imported into software for further analysis. The landscape of data processing is evolving rapidly, however, with significant advancements in real-time visualization and reconstruction directly from data streams. This shift is driven by the escalating challenge of data management; as the volume of data generated outpaces the capacity for traditional storage and processing methods, the efficiency of saving to disk and subsequently transferring data to high-performance computing (HPC) resources emerges as a critical bottleneck. For example, the tomoscopy setup at PSI can acquire data at acquisition rates of up to 1000 tomograms per second (García-Moreno *et al.*, 2021 ▸). To deal with this, packages such as *RECAST3D* (Schoonhoven *et al.*, 2020 ▸) and *tomoStream* (Nikitin *et al.*, 2022 ▸) enable on-the-fly processing of the data streams, dramatically reducing turnaround times.

## 3. *TomoPyUI*: a graphical interface for *TomoPy*

**Figure 1:**
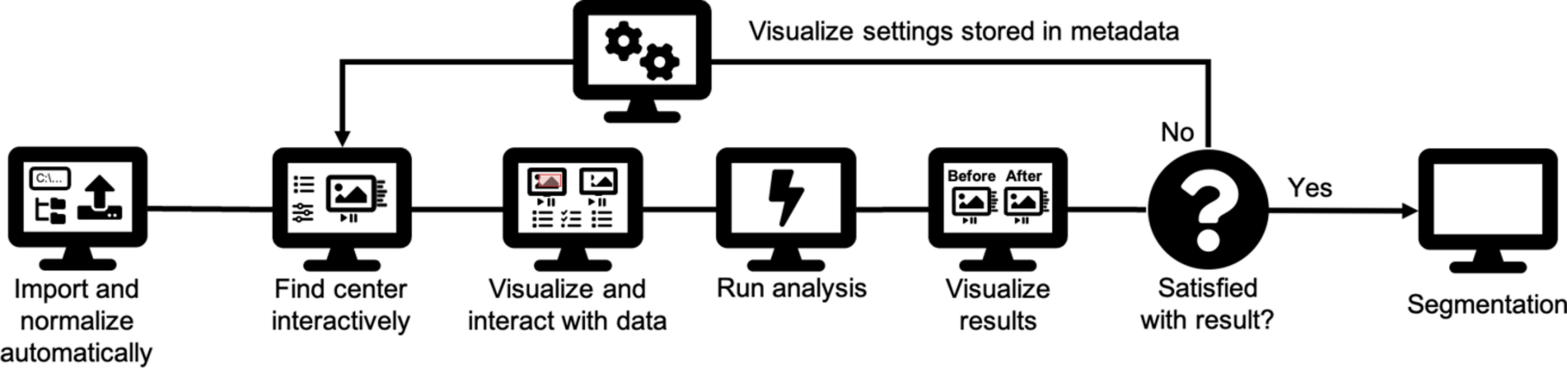
*TomoPyUI* processing flowchart. The intuitive user interface of *TomoPyUI* allows users to move through the tomography processing pipeline in a streamlined manner. Figure created using FontAwesome Free icons, creative commons license CC BY 4.0 (https://fontawesome.com/license/free).

Within the dynamically evolving landscape of data management solutions, *TomoPyUI* stands out as a platform that not only facilitates a fully interactive experience throughout the processing pipeline, but also enables access to remote data and computational resources. The interactive experience begins when raw projection data are imported into *TomoPyUI*. These data are automatically normalized and displayed in an image viewer, along with acquisition information (*e.g.* number of projection images, image size, X-ray energy *etc.*) in the form of a metadata table, providing accessible and contextual information about the imported data. The user can then work through the rest of the data processing pipeline interactively and visually, applying methods developed in *TomoPy* to their data (Fig. 1 ▸). The pipeline currently includes image filtration, center of rotation tools, and automatic alignment and reconstruction. Metadata are saved at each step of this process, tracking the processing performed and allowing the user to pick up where they left off. Additionally, the user has the flexibility to return to any stage along the pipeline and redirect their analysis if so desired. Some of the features available in other software (*e.g.* 3D XANES analysis) are not currently implemented in *TomoPyUI* but could be added; the software is open-source and is structured in a manner that lends itself to feature addition, which is discussed later (see *Program Structure*[Sec sec6]).

Developed within the *Jupyter* ecosystem, *TomoPyUI* capitalizes on the ability of the *Jupyter* programming environment to run on remote hardware. This is a strategic response to the evolving challenges in data management and analysis at light source facilities, which are poised to generate an exabyte of data per year by 2028 (Schwarz *et al.*, 2020 ▸). Notably, premier HPC centers, such as NERSC at Lawrence Berkeley National Laboratory, have embraced *Jupyter* as a core programming environment (Thomas & Cholia, 2021 ▸), underscoring its significance. Although existing tools like *Savu* and *tomwer* provide mechanisms for remote operations, their integration into a web interface remains limited. *Tomwer*, for instance, supports remote access, but its setup demands more from the user (*i.e.* setting up X-forwarding) compared with the intuitive approach of running a cell in a *Jupyter* notebook. By integrating directly with the *Jupyter* ecosystem, *TomoPyUI* simplifies the process of engaging with HPC resources by allowing users to initiate the application on a remote *Jupyter­Hub*. Further, while *TomoPyUI* does not currently offer a way to visualize processed data streams from instruments, its tools built in the *Jupyter* ecosystem could be extended to enable real-time visualization.

## 4. Key features of *TomoPyUI*

This section highlights the main features of *TomoPyUI*. It is not intended to explain all the tabs in *TomoPyUI* step-by-step; see the video tutorials and walkthroughs at https://tomopyui.readthedocs.io/.

### 4.1. Pyramidal downsampling

*TomoPyUI* relies on both full-resolution and downsampled data. Whenever data are imported, they are downsampled pyramidally using *dask* (Rocklin, 2015 ▸). If a dataset contains 360 2048 × 2048 images, *TomoPyUI* will create additional datasets containing 360 1024 × 1024 images, 360 512 × 512 images and so on. These secondary datasets are stored on the same hdf5 file as the full-resolution dataset. Downsampling serves two crucial functions within *TomoPyUI*: reduced lag time in refreshing changes to the displayed output image and facilitating fast alignment.

**Figure 2:**
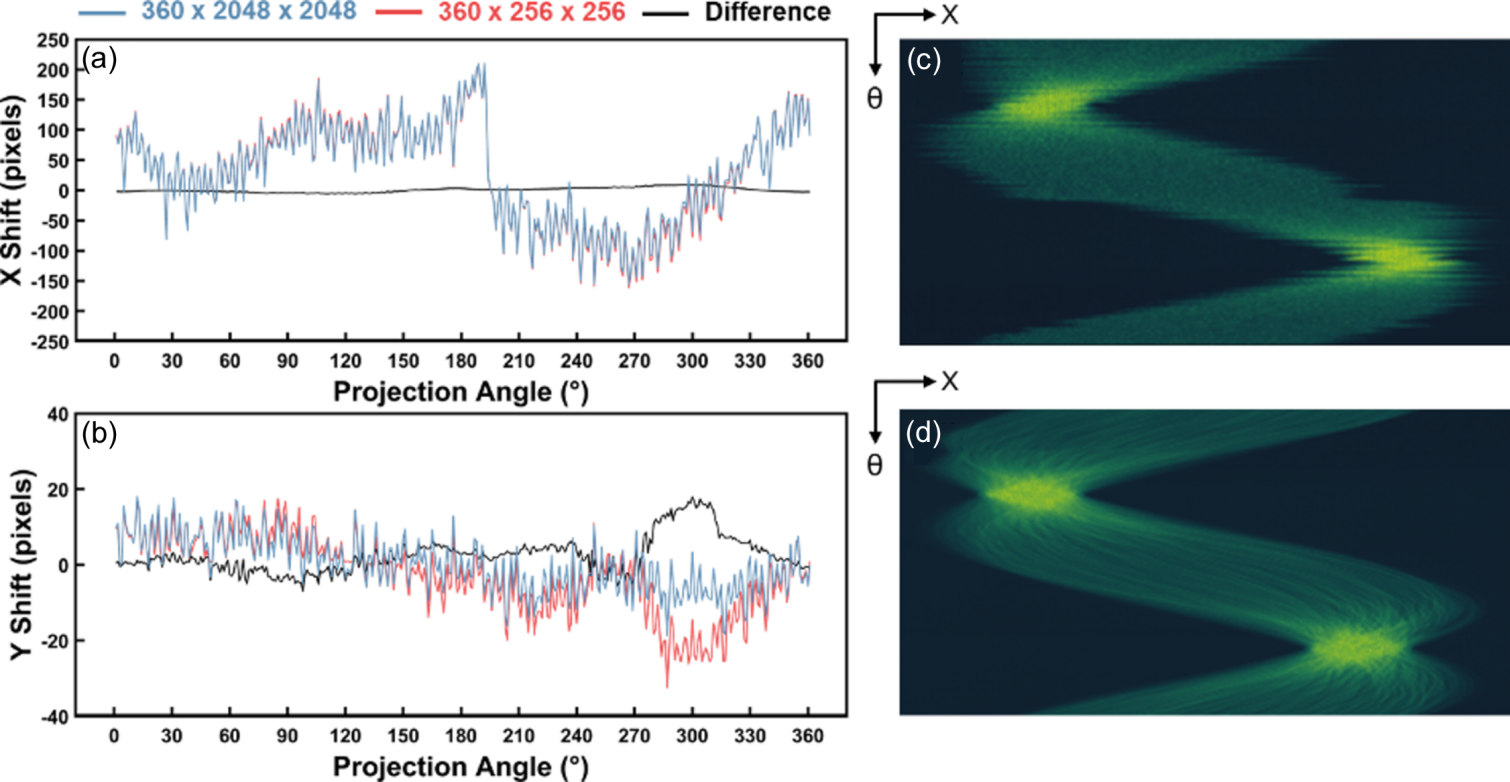
Impact of downsampling data on alignment efficacy. (*a*) *X* pixel shift and (*b*) *Y* pixel shift with respect to the projection angle (θ) for both full resolution (360 × 2048 × 2048) in blue and downsampled (360 × 256 × 256) in red datasets. Example of sinogram (*c*) before and (*d*) after automatic alignment using downsampled data. The downsampled dataset took a nearly a fifth of the time that the full dataset took to align.

The alignment protocol incorporated into *TomoPy* and *TomoPyUI* (joint iterative reconstruction and reprojection) (Gürsoy *et al.*, 2017 ▸) can be computationally time intensive when full-resolution datasets are used. In addition, full-resolution data contain Poisson noise, which can be partially filtered by downsampling to improve the efficacy of the alignment algorithm (Gürsoy *et al.*, 2017 ▸; Marais & Willett, 2017 ▸). In many cases, using downsampled data produces similar, if not identical, alignment results compared with the full-resolution data. To demonstrate this, we took 360 projection images of a nanoporous gold sample at SSRL beamline 6-2c in 1° increments and used *TomoPyUI* to align both the full-resolution dataset (360 × 2048 × 2048) and a heavily downsampled dataset (360 × 256 × 256). Figs. 2 ▸(*a*) and 2 ▸(*b*) show the *X* and *Y* pixel shifts, respectively, following 300 iterations of automated alignment, utilizing simultaneous iterative reconstruction technique (SIRT) as the reconstruction algorithm. Note that the iterative alignment process depends on cross-correlation between the initial projection images and the images re-projected from the reconstruction. Consequently, the selection of the reconstruction algorithm influences the outcome of the alignment. The blue line represents the shift of the full-resolution dataset, the red line represents the downsampled dataset shifts, and the black line represents the difference between these shifts. Despite only slight deviations from 0 in the shift difference, the total alignment time was 4.8 times longer for the full-resolution dataset than for the downsampled dataset. Considering the time difference and the robustness of the alignment, we recommend using the downsampled data to perform an initial alignment. After the alignment is completed, two image viewers appear to compare projection images and sinograms before and after the alignment [Figs. 2 ▸(*c*) and 2 ▸(*d*)]. Based on visual inspection, the user can choose to use the aligned data for another alignment at a higher resolution (*i.e.* less downsampling), or try another alignment with different options using the original dataset.

Note that *TomoPyUI* also works with larger datasets typical of microCT. Please see the supporting information for a demonstration of a round-robin dataset from *TomoBank* (De Carlo *et al.*, 2018 ▸).

### 4.2. Image viewers

**Figure 3:**
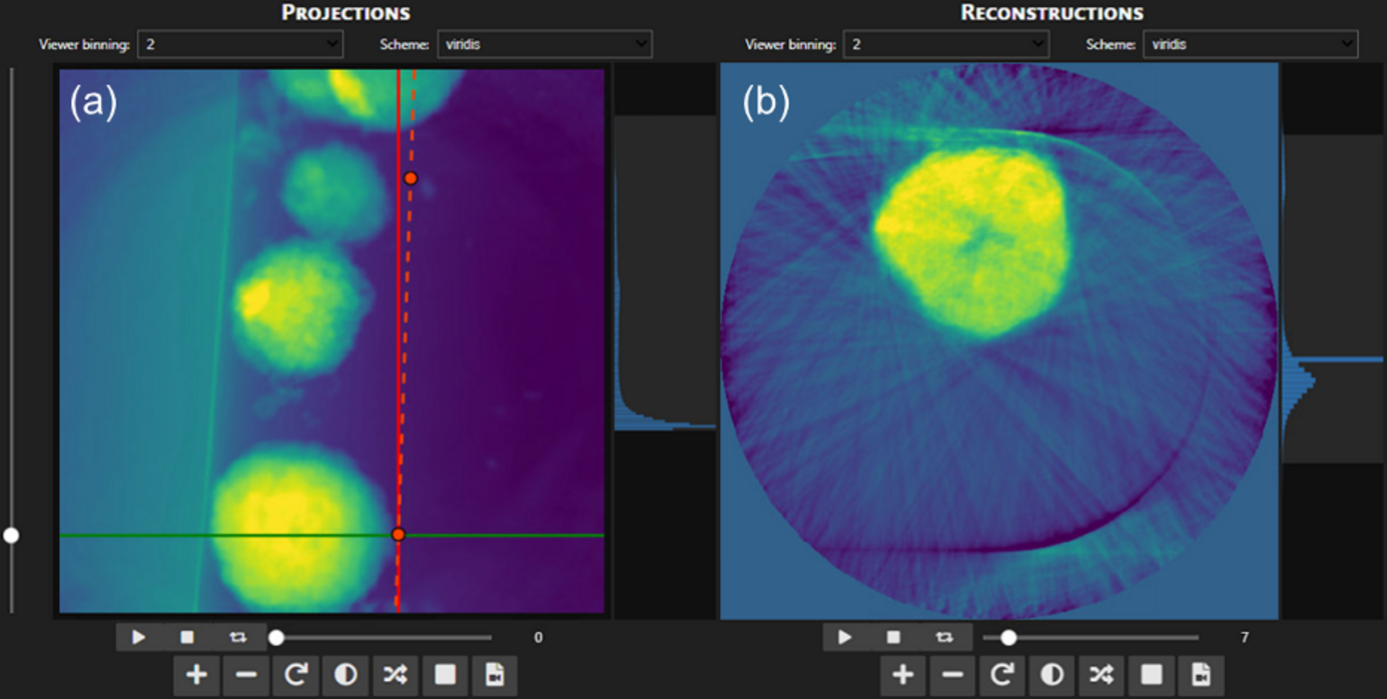
Image viewers in the *Center* tab. Using the vertical slider on the left side of the *Projections* viewer (*a*) one chooses a slice to reconstruct. After choosing options for the center of rotation range (not shown), the reconstructed slice over a range of different centers is plotted in the (*b*) *Reconstructions* image viewer. One can quickly check the results of different centers by sliding through the reconstructed slices. If the rotation axis has a tilt, users can set multiple center points to create a list of centers that varies linearly along the slice dimension [orange dashed line in (*a*)].

Another key feature of *TomoPyUI* is its built-in, modular image viewer in each tab of the application for visualizing, manipulating and interacting with data. For example, in the *Center* tab, we provide a center of rotation-finding wizard that is modeled from the *tomopy* center of rotation search found in the *tomopy.recon.rotation* module (Fig. 3 ▸).

The user can quickly choose a slice to reconstruct [green horizontal line, Fig. 3 ▸(*a*)] using the vertical slider [left, Fig. 3 ▸(*a*)] and hence reconstruct that slice for a given range of rotation centers. This uses a wrapper around *tomopy.recon.rotation.write_center*, where instead of writing the reconstructed slices to disk, it stores them in the *Reconstructions* image viewer [Fig. 3 ▸(*b*)]. After reconstruction, they can determine the best center by sliding through the reconstructed slice for the range of centers. The center value is automatically updated in the *Alignment*/*Reconstruction* tabs to ensure a smooth transition between processing steps. To accommodate scenarios where projection images rotate around a non-perpendicular axis, one can iteratively define multiple centers of rotation at different slices of the dataset. This is achieved by setting several centers [marked as red points in Fig. 3 ▸(*a*)] by repeating the process outlined above at various slices. We point the reader to our online documentation for a video explanation of this process (https://tomopyui.readthedocs.io/).

**Figure 4:**
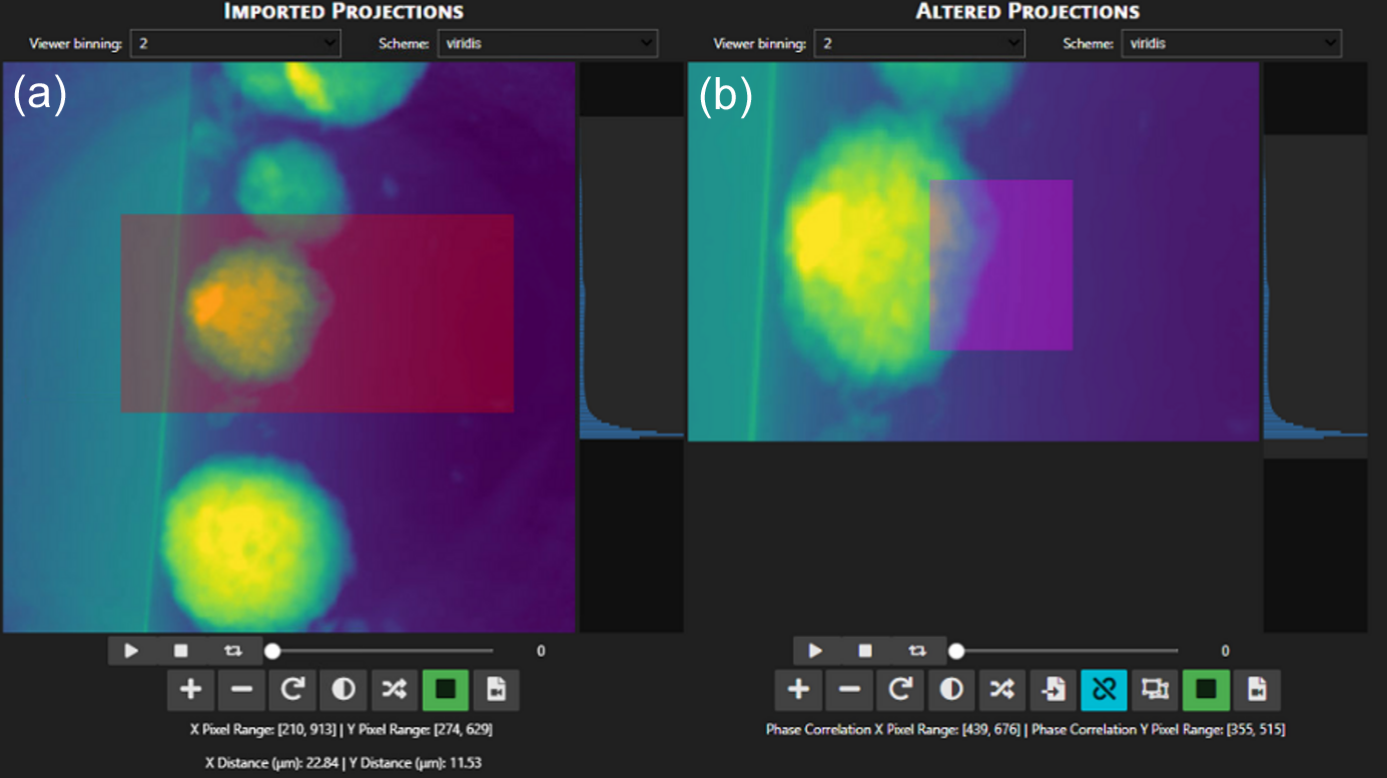
Image viewers displayed in the alignment and reconstruction tabs. (*a*) *Imported Projections* viewer, where an ROI shown in red captures a portion of the data to send to the (*b*) *Altered Projections* viewer, which is the data used for alignment or reconstruction. The *Altered Projections* viewer ROI is used to select a pixel range to compare the projected and reprojected images in the joint iterative alignment algorithm (Gürsoy *et al.*, 2017 ▸).

In the *Align* and *Reconstruct* tabs, users can select a region of interest (ROI) within their dataset in the *Imported Projections* viewer (red box, Fig. 4 ▸). This selection is then transferred to the *Altered Projections* viewer, which will then be used for computation of alignment and/or reconstruction. The ability to reduce the dataset in this way saves alignment and reconstruction time, and it can dramatically improve the efficacy of the joint iterative alignment scheme. For example, projection image datasets may have objects that float into the field of view for a small fraction of the projection angles; cropping these objects out can improve alignment.

## 5. Example alignment and reconstruction

In the following section, we demonstrate the efficacy of the *TomoPyUI* automatic alignment algorithm using a real dataset.

### 5.1. Experimental

Particles from a cycled Li-ion pouch cell (LiNi_0.5_Mn_0.3_Co_0.2_O_2_) were loaded into a 0.3 mm inner diameter glass capillary. A total of 1800 projection images (1024 × 1024 pixels) were acquired on beamline 6-2c at the Stanford Synchrotron Radiation Lightsource over 180° with angular resolution of 1° at 8353 eV. Ten projection images were averaged at each angle, and reference images were taken every 10°. For more experimental details, refer to Preefer *et al.* (2022 ▸).

### 5.2. Computed tomography before and after various alignment protocols

**Figure 5:**
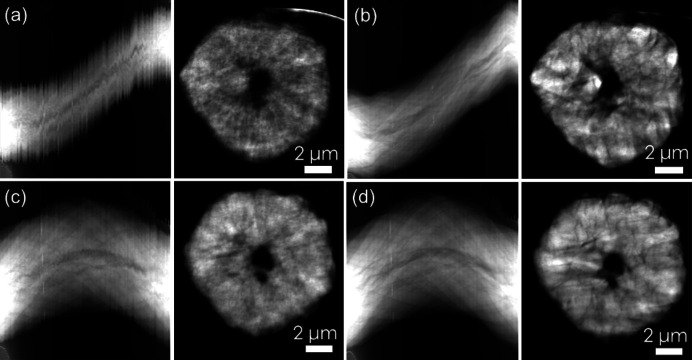
Sinograms (left image of each panel) and *XY* slice of reconstructions (right image of each panel) of a Li-ion battery cathode particle with various alignment protocols. (*a*) Unaligned; (*b*) automatically aligned using *TomoPyUI*; (*c*) manually aligned using *TXM Wizard*; and (*d*) manually aligned using *TXM Wizard*, then automatically aligned using *TomoPyUI.*

To demonstrate the effectiveness of alignment in *TomoPyUI*, we show a sinogram and a reconstruction slice for several different methods of alignment in Fig. 5 ▸. The discontinuities visible in the unaligned sinogram in Fig. 5 ▸(*a*) indicate a significant amount of jitter in the raw dataset. The jitter appreciably impacts the quality of reconstruction: nanoscale features are completely blurred out. On the other hand, after two short (18 s) alignments using a heavily downsampled dataset in *TomoPyUI* [Fig. 5 ▸(*b*)], the overall shape of the sinogram has not been impacted, but the discontinuities (jitter) have been removed and thus the quality of reconstruction has dramatically improved. It is evident that the central hole in the particle comprises a larger and a smaller hole, and smaller cracks can be seen throughout the particle. Fig. 5 ▸(*c*) is the sinogram and resulting reconstruction slice of the raw data in Fig. 5 ▸(*a*) that have been manually aligned in *TXM Wizard*. During manual alignment in *TXM Wizard*, the user clicks the same point on an object in each of the projection images, shifting the image from that point to the center *X* and *Y* pixels. Even if the object has a non-zero transverse displacement from the rotation axis of the sample holder, it is placed on the center of rotation in the images, altering the sinograms’ shape [compare the sinograms in Fig. 5 ▸(*a*) with those in Fig. 5 ▸(*c*)]. Some jitter still persists in the manually aligned data in Fig. 5 ▸(*c*), and while the resolution of the resulting reconstruction slice is better than the raw reconstruction in Fig. 5 ▸(*a*) (*e.g.* smaller cracks throughout the particle can be observed), the automatically aligned reconstruction exhibits better feature resolution. Finally, we automatically aligned the manually aligned dataset in Fig. 5 ▸(*c*) to further remove jitter which, again, significantly improves feature visibility in the reconstruction. Although it appears that Fig. 5 ▸(*d*) has a comparable resolution to Fig. 5 ▸(*b*), the overall particle shape in the reconstructed slice in Fig. 5 ▸(*b*) more closely resembles that in the reconstructed slice in Fig. 5 ▸(*a*). Note the particle perimeter and the shape of the large hole in the particle center. We speculate that the manual alignment, which sets the rotation center to the *X* value of the point clicked by the user in the image, impacts the particle shape. However, since we do not have a ground truth measurement for this particle, we cannot determine which methodology is better: automatically aligned [Fig. 5 ▸(*b*)] or manually and then automatically aligned [Fig. 5 ▸(*d*)]. Moreover, the best methodology is likely to be sample dependent. For example, the shape of a particle close to the center of rotation may be less impacted by manual alignment than the example given here. Nevertheless, using *TomoPyUI* to automatically align a downsampled dataset twice (a total of 36 s of computation time) is far more desirable for the user than iteratively clicking 180 images and makes higher angular resolution data reasonable to collect and align. In some cases (*i.e.* when objects that float into the field of view cannot effectively be removed by cropping), performing a manual alignment prior to automatic alignment may be necessary to avoid automatic alignment failure.

## 6. Program structure

### 6.1. Overview

**Figure 6:**
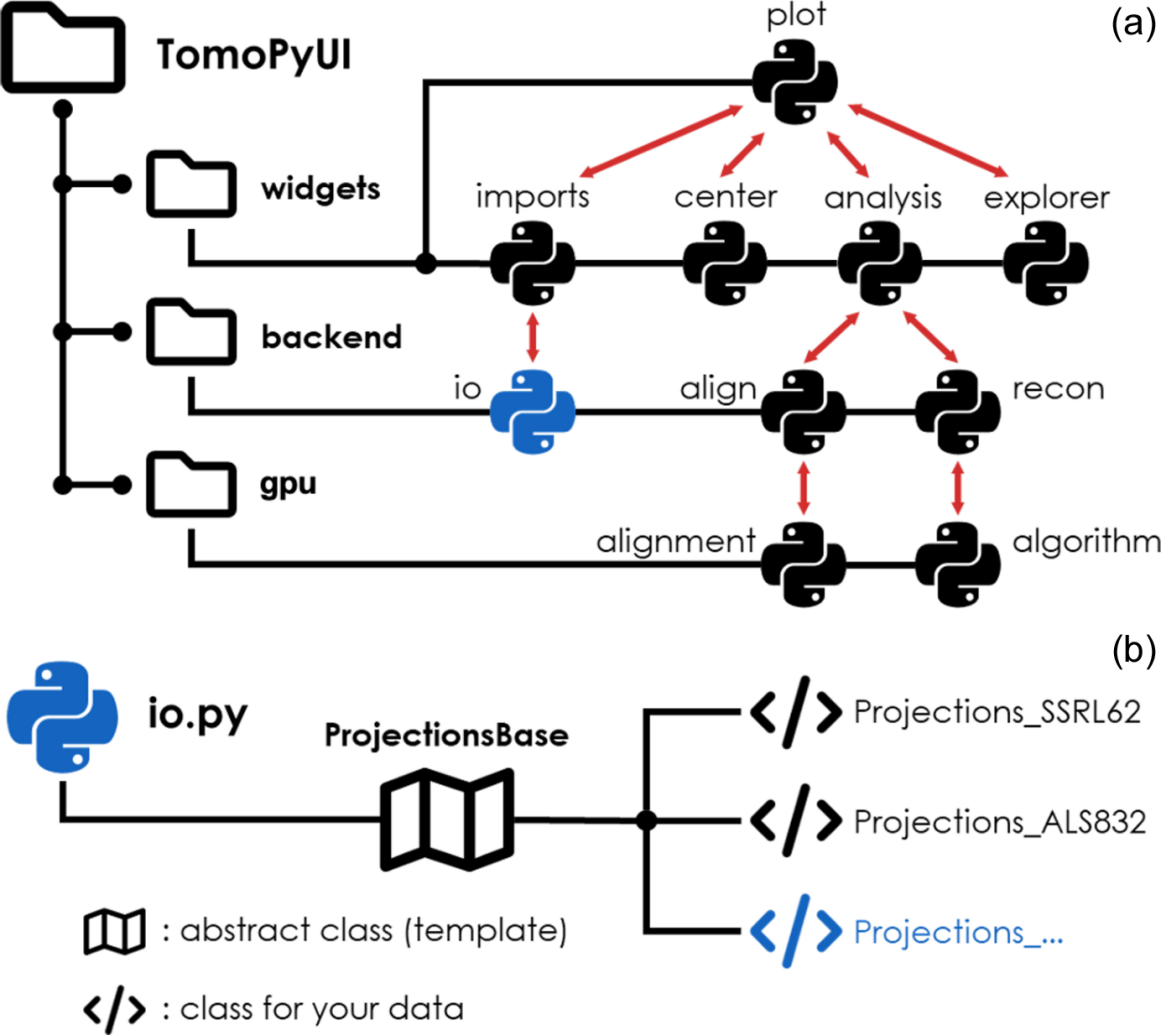
(*a*) Structure of *TomoPyUI*. The submodules of the code interact (shown by the red arrows) to create the GUI. The Python symbols indicate individual Python files, and the black lines indicate their subfolder (*i.e.* widgets, backend and gpu). (*b*) Scalability to other beamlines. Only minor modifications need to be made to a selection of the Python files (*e.g.* io.py shown in blue) to add import functionality for specific beamlines. Figure created using FontAwesome Free icons, creative commons license CC BY 4.0 (https://fontawesome.com/license/free).

*TomoPyUI* is an open-source GUI, built entirely in Python, with its frontend operating within the *Jupyter* ecosystem (Kluyver *et al.*, 2016 ▸). The project uses several actively developed Python packages, including *ipywidgets*, *bqplot*, *TomoPy*, *dask*, *Astra Toolbox* and *cupy*. An overview of the structure of the project is shown in Fig. 6 ▸(*a*), containing three modules: (1) *widgets*, which houses the user interface and is built primarily from *bqplot*, *bqplot-image-gl* and *ipywidgets*; (2) *backend*, where data and metadata input/output and alignment/reconstruction functions run and is built using *TomoPy* and *dask*; and (3) *gpu*, a Compute Unified Device Architecture (CUDA)-enhanced alignment and reconstruction package that runs significantly faster than the CPU based *TomoPy* routines by leveraging the power of GPUs and making use of *cupy* and *Astra Toolbox*. For each step of the data processing pipeline, there is a *widgets* submodule that creates the corresponding frontend (GUI) tab, including data import in *imports*, center of rotation search in *center*, and alignment and reconstruction in *analysis*. Each of these submodules converses with the *view* submodule, which contains Python classes that create the image visualizers present in each tab and discussed above. After choosing various options in the frontend, *backend* and *gpu* perform the behind-the-scenes computations and send the result to the frontend on completion. Many of the tools in *backend* and *gpu* are wrappers for *TomoPy* functions with enhanced features, including real-time visualization during alignment and algorithms sped up by CUDA [calling the *Astra Toolbox* (van Aarle *et al.*, 2016 ▸) directly and using *cupy*]. Note that while these backend enhancements currently reside within the *TomoPyUI* codebase, they could be integrated into *TomoPy* (Gürsoy *et al.*, 2014 ▸) and/or *TomocuPy* (Nikitin, 2023 ▸) in the future to make these features more widely accessible.

### 6.2. Modularity and object-oriented framework

While developing *TomoPyUI*, we focused on code modularity in three of its key features such that it could be scaled to other beamlines and provide the backbone for other 3D synchrotron data software.

(1) *Data import*. Since each of the CT beamlines stores raw data in different file formats, software logic behind data import can be burdensome (De Carlo *et al.*, 2014 ▸). For example, it can be challenging to create a general import function that could detect whether an .h5 file is from the Advanced Light Source (ALS) or Advanced Photon Source (APS). On top of this, the normalization procedure of one beamline may differ from another. Projection images collected at the SSRL beamline 6-2c, for example, are saved as a series of darkfield-corrected images that need to be averaged together after normalization. Other beamlines may store pre-averaged projection images and darkfield images separately in an hdf5 file. Given these complications, we created a class inheritance structure to enable new import protocols to be deployed in *TomoPyUI* with relative ease. We have provided a guide to create a new import protocol through an example pull request (https://github.com/swelborn/tomopyui/pull/41) for the import of raw data stored in the NXTomo format file. The current version of *TomoPyUI* requires datasets to fit in the memory of the host machine during import. Note, however, that one could implement their own data import protocol that mitigates this requirement.

(2) *Metadata management*. Similar to our data import class structure, *TomoPyUI* contains metadata classes (subclasses of the metadata class in *io.py*) that manage metadata storage both during import and throughout the processing pipeline, allowing for quick and easy visual access to this information in the form of metadata tables displayed on the *Import* tab. Each type of metadata has its own metadata class, and thus the table may appear different for data from different beamlines. While only a few metadata variables are required for data import for *TomoPyUI* to function properly (*e.g.* start and end angles, reference location indices, and pixel size), additional experiment- or beamline-specific values can be added to inform the user about their acquisition.

(3) *Data visualization*. The data visualizers in each tab of *TomoPyUI* are constructed from the various viewer classes found in *view.py*, built using a combination of widgets from the Python packages *bqplot*, *bqplot-image-gl* and *ipywidgets*. These viewers all contain the same base-level functionality including an image histogram, an image index slider and several common image visualization buttons. However, additional and distinct functionality is required in each tab (*e.g.* an additional slider for finding the centers of rotation). For this reason, we created a subclass structure that lends itself to easily building these features on top of the image viewer base class. We should emphasize that our data visualization logic could be applied to other image series data without significant changes to our modular, open-source code.

## 7. Conclusions

This manuscript presents *TomoPyUI*, a GUI that builds on the *TomoPy* library to facilitate improved interaction with computed tomography (CT) data. *TomoPyUI* responds to the growing needs for data management and analysis capabilities at synchrotron and neutron facilities. *TomoPyUI* leverages the ability of the *Jupyter* ecosystem to operate on remote hardware, thereby making advanced computing resources more accessible to a wider range of users. The utilities we have developed in *TomoPyUI* enable these users to navigate the processing pipeline efficiently by interactively executing tasks such as identifying the center of rotation and aligning projection images, facilitating rapid processing feedback loops. Additionally, *TomoPyUI* supports the storage and visualization of metadata, enabling users to easily monitor the modifications and processing steps applied to their data within the workspace. As an open-source project (https://github.com/swelborn/tomopyui), it encourages contributions and feedback from the user community to ensure it meets the evolving needs of researchers in the field. For instructions on how to install *TomoPyUI*, import data, and utilize its alignment and reconstruction features, users are encouraged to consult the documentation and tutorials available at https://tomopyui.readthedocs.io/.

## Supplementary Material

Supporting figures. DOI: 10.1107/S1600577524003989/vl5020sup1.pdf
