# Supplementary material for: TomoPyUI: a user-friendly tool for rapid tomography alignment and reconstruction
Source: J Synchrotron Radiat. 2024 Jun 26;31(Pt 4):979–86. doi: 10.1107/S1600577524003989 (PMC11226142; doi:10.1107/S1600577524003989)
Supplement: Supplementary file 1 [file s-31-00979-sup1.pdf]

JOURNAL OF
SYNCHROTRON
RADIATION

Volume 31 (2024)

Supporting information for article:

TomoPyUI: a user-friendly tool for rapid tomography alignment and reconstruction

Samuel S. Welborn, Molleigh B. Preefer and Johanna Nelson Weker

Supplemental Information to TomoPyUI: a User-friendly Tool for Rapid Tomography Alignment and Reconstruction

Samuel S. Welborn,[1,2] Molleigh B. Preefer,[2] Johanna Nelson Weker[2]

[1]*Department of Materials Science & Engineering, University of Pennsylvania, Philadelphia, Pennsylvania 19104, USA.*
[2]*Stanford Synchrotron Radiation Lightsource, SLAC National Accelerator Laboratory, Menlo Park, California 94025, USA.*
**Correspondence should be addressed to J.N.W. (jlnelson@slac.stanford.edu)*

Handling larger datasets

TomoPyUI is able to handle the import and processing of large datasets. For an example of this, we have imported the tomo_00001 (from the round-robin datasets) from tomobank[1] into *TomoPyUI* using the APS_32ID importer on this GitHub pull request. This dataset, approximately 11 GB in size, comprises around 1500 projections with dimensions of 1792 x 2048 pixels.

In **Supplemental Figure 1**, we show that importing the data and finding its center of rotation is possible within *TomoPyUI*. In **Supplemental Figure 2**, we show that this data can be used for alignment. Here we have used performed 10 iterations of the joint alignment/reconstruction algorithm using the SIRT3D *Astra* algorithm on a subset of the full dataset (8x binning, pixel range shown in "Analysis Projections").

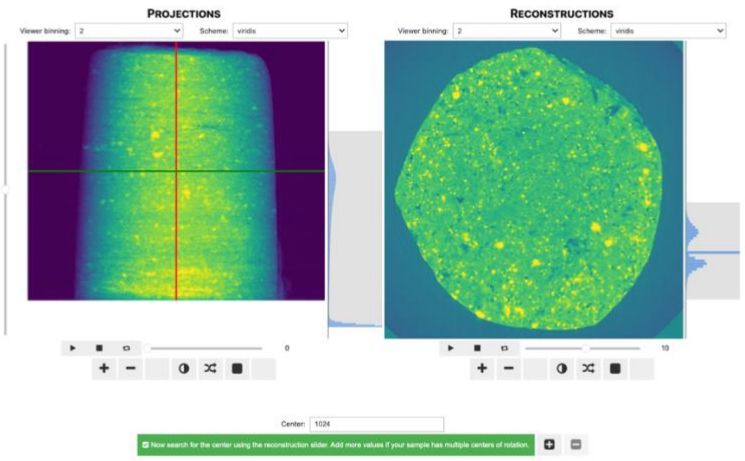

Supplemental Figure 1. Identification of the center of rotation within *TomoPyUI* for the tomo_00001 dataset from TomoBank.

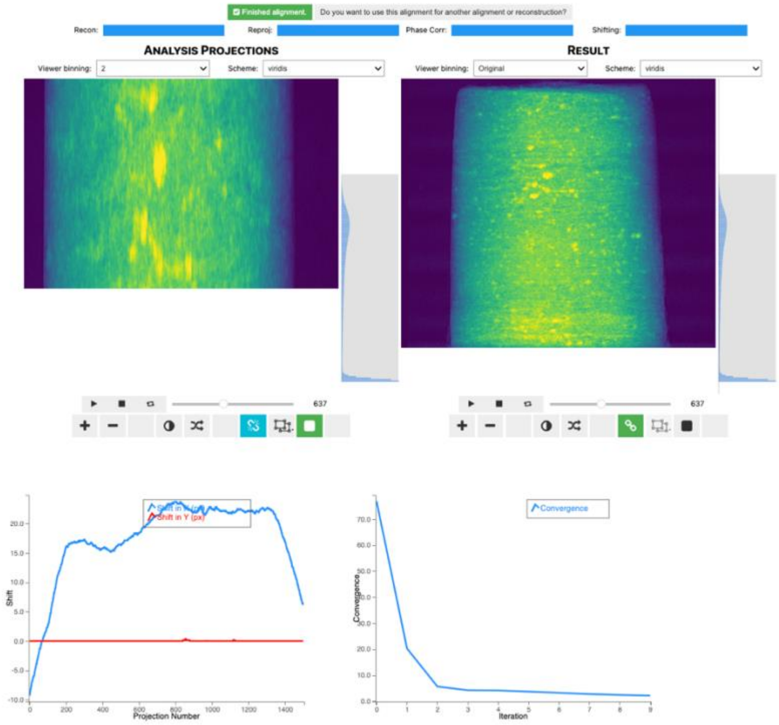

Supplemental Figure 2. Alignment of the tomo_00001 dataset after conducting 10 iterations of the joint alignment/reconstruction method using the SIRT3D algorithm from Astra on a downsampled subset (8x binning). The "Analysis Projections" details the pixel range involved in the alignment, showcasing *TomoPyUI*'s capability to use only a fraction of the full dataset for alignment.
